# Supplementary material for: Predicting visual function by interpreting a neuronal wiring diagram
Source: Nature. 2024 Oct 2;634(8032):113–23. doi: 10.1038/s41586-024-07953-5 (PMC11446822; doi:10.1038/s41586-024-07953-5)
Supplement: Supplementary file 6 — CRF and ERF predictions for individual TmY4 and TmY9 cells. Analogous to Supplementary Data 3, but for TmY target types. Shown are the top four monosynaptic pathways, the strongest pathway passing through each of the top ten intermediary types (ranking from Extended Data Fig. 7), and the trisynaptic pathway Tm1–TmY–Dm3–TmY (see the section entitled Prediction of spatial normalization). [file 41586_2024_7953_MOESM6_ESM.zip › DataS4/TmY9q/720575940627940883.pdf]

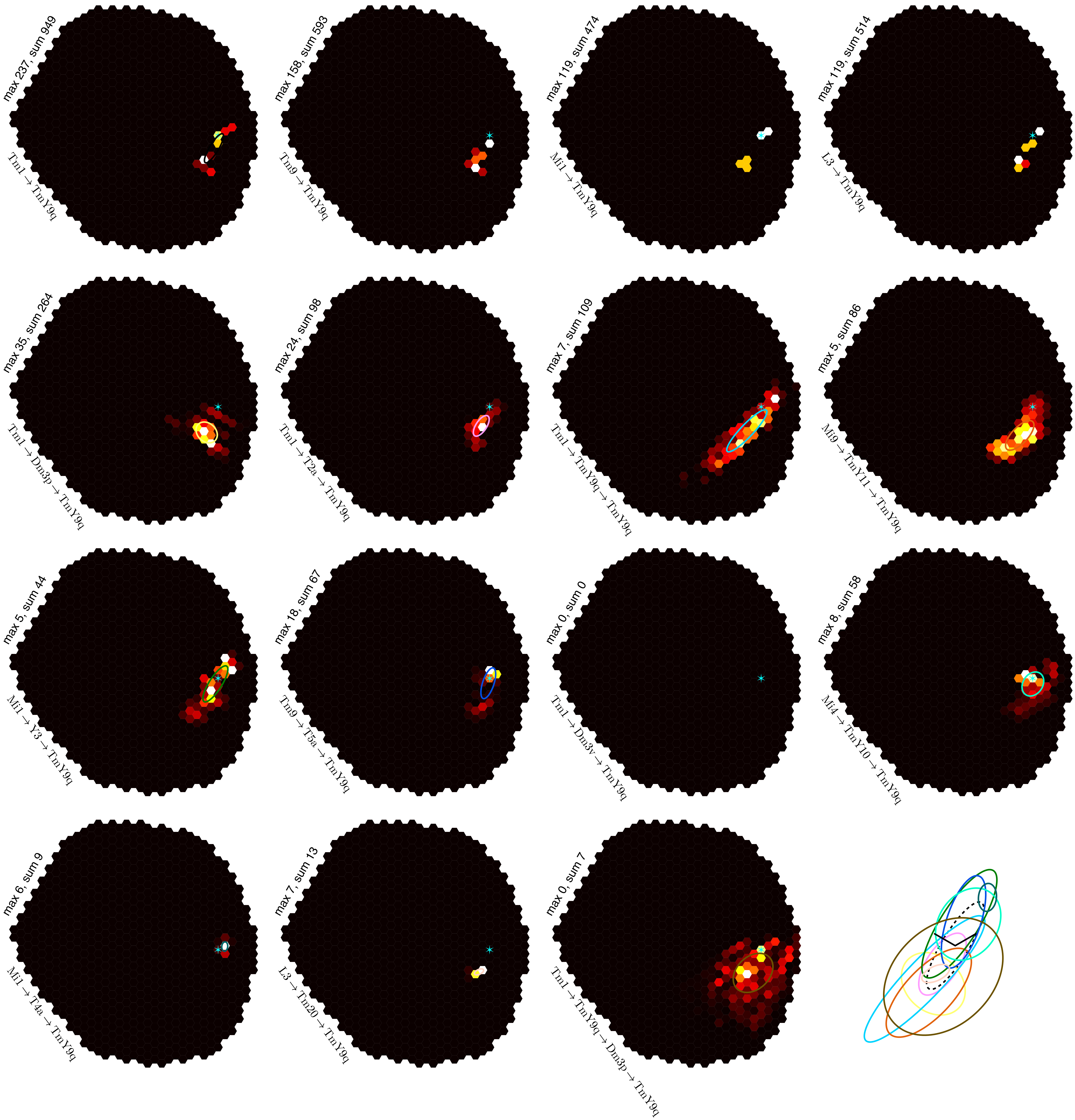

max 237, sum 949
Tm1 → TmY9q
max 158, sum 593
Tm9 → TmY9q
max 119, sum 474
Mi1 → TmY9q
max 119, sum 514
L3 → TmY9q
max 35, sum 264
Tm1 → Dm3p → TmY9q
max 24, sum 98
Tm1 → T2a → TmY9q
max 7, sum 109
Tm1 → TmY9q → TmY9q
max 5, sum 86
Mi9 → TmY11 → TmY9q
max 5, sum 44
Mi1 → Y3 → TmY9q
max 18, sum 67
Tm9 → T5a → TmY9q
max 0, sum 0
Tm1 → Dm3v → TmY9q
max 8, sum 58
Mi4 → TmY10 → TmY9q
max 6, sum 9
Mi1 → T4a → TmY9q
max 7, sum 13
L3 → Tm20 → TmY9q
max 0, sum 7
Tm1 → TmY9q → Dm3p → TmY9q
